# Association between Polyphenol Intake and Breast Cancer Risk by Menopausal and Hormone Receptor Status

**DOI:** 10.3390/nu12040994

**Published:** 2020-04-03

**Authors:** Facundo Vitelli-Storelli, Raul Zamora-Ros, Antonio J. Molina, Tania Fernández-Villa, Adela Castelló, Juan Pablo Barrio, Pilar Amiano, Eva Ardanaz, Mireia Obón-Santacana, Inés Gómez-Acebo, Guillermo Fernández-Tardón, Ana Molina-Barceló, Juan Alguacil, Rafael Marcos-Gragera, Emma Ruiz-Moreno, Manuela Pedraza, Leire Gil, Marcela Guevara, Gemma Castaño-Vinyals, Trinidad Dierssen-Sotos, Manolis Kogevinas, Nuria Aragonés, Vicente Martín

**Affiliations:** 1 Group of Investigation in Interactions Gene-Environment and Health (GIIGAS)/Institute of Biomedicine (IBIOMED), Universidad de León, 24071 León, Spain; fvits@unileon.es (F.V.-S.); ajmolt@unileon.es (A.J.M.); tferv@unileon.es (T.F.-V.); jpbarl@unileon.es (J.P.B.); vicente.martin@unileon.es (V.M.); 2 Unit of Nutrition and Cancer, Cancer Epidemiology Research Programme, Catalan Institute of Oncology (ICO), Bellvitge Biomedical Research Institute (IDIBELL), L’Hospitalet del Llobregat, 08908 Barcelona, Spain; 3 School of Medicine, University of Alcalá, 28871 Alcalá de Henares, Madrid, Spain; acastello@externos.isciii.es; 4 Consortium for Biomedical Research in Epidemiology & Public Health (CIBER Epidemiología y Salud Pública—CIBERESP), 28029 Madrid, Spain; epicss-san@euskadi.eus (P.A.); me.ardanaz.aicua@navarra.es (E.A.); ines.gomez@unican.es (I.G.-A.); fernandeztguillermo@uniovi.es (G.F.-T.); alguacil@dbasp.uhu.es (J.A.); rafael.marcos@udg.edu (R.M.-G.); e.ruiz@externos.isciii.es (E.R.-M.); l-gil@euskadi.eus (L.G.); mp.guevara.eslava@navarra.es (M.G.); gemma.castano@isglobal.org (G.C.-V.); trinidad.dierssen@unican.es (T.D.-S.); manolis.kogevinas@isglobal.org (M.K.); nuria.aragones@salud.madrid.org (N.A.); 5 Public Health Division of Gipuzkoa, BioDonostia Research Institute, 20014 San Sebastian, Spain; 6 Public Health Institute of Navarra, IdiSNA, 31003 Pamplona, Spain; 7 Oncology Data Analytics Program (ODAP), Catalan Institute of Oncology (ICO), L’Hospitalet del Llobregat, 08908 Barcelona, Spain; mobon@idibell.cat; 8 ONCOBELL Program, Bellvitge Biomedical Research Institute (IDIBELL), L’Hospitalet de Llobregat, 08908 Barcelona, Spain; 9 Consortium for Biomedical Research in Epidemiology and Public Health (CIBERESP), 28029 Madrid, Spain; 10 Oncology Institute, University of Oviedo, 33003 Oviedo, Spain; 11 Cancer and Public Health Area, FISABIO—Public Health, 46035 Valencia, Spain; molina_anabar@gva.es; 12 Centro de Investigación en Salud y Medio Ambiente (CYSMA), Universidad de Huelva, Campus Universitario de El Carmen, 21071 Huelva, Spain; 13 Catalan Institute of Oncology, Epidemiology Unit and Girona Cancer Registry, Oncology Coordination Plan, Department of Health, Autonomous Government of Catalonia, Catalan Institute of Oncology, 17007 Girona, Spain; 14 Descriptive Epidemiology, Genetics and Cancer Prevention Group, Biomedical Research Institute (IDIBGI), 17090 Girona, Spain; 15 Research Group on Statistics, Econometrics and Health (GRECS), University of Girona, 17004 Girona, Spain; 16 National Center for Epidemiology, Carlos III Institute of Health, 20014 San Sebastián, Spain; 17 Department of Oncology, Complejo Asistencial Universitario de León, 24071 León, Spain; maitapedraza@hotmail.com; 18 Biodonostia Health Research Institute, 20013 San Sebastian, Spain; 19 ISGlobal, Barcelona, 08036 Barcelona, Spain; 20 IMIM (Hospital del Mar Medical Research Institute), 08003 Barcelona, Spain; 21 Universitat Pompeu Fabra (UPF), Campus del Mar, 08003 Barcelona, Spain; 22 Universidad de Cantabria—IDIVAL, 39011 Santander, Spain; 23 Epidemiology Section, Public Health Division, Department of Health of Madrid, 28035 Madrid, Spain

**Keywords:** flavonoids, polyphenols, classes, intake, breast cancer, case-control

## Abstract

There is limited evidence of phenolic compounds acting as protective agents on several cancer types, including breast cancer (BC). Nevertheless, some polyphenol classes have not been investigated and there is a lack of studies assessing the effect on menopausal status and hormone receptor status as influenced by these compounds. The objective of this study is to evaluate the association between the intake of all polyphenol classes in relation to the BC risk by menopausal and hormone receptor status. We used data from a population-based multi-case-control study (MCC-Spain) including 1472 BC cases and 1577 controls from 12 different regions of Spain. The odds ratios (ORs) with 95% CI were calculated using logistic regression of mixed effects by quartiles and log2 of polyphenol intakes (adjusted for the residual method) of overall BC, menopausal and receptor status. No associations were found between total intake of polyphenols and BC risk. However, inverse associations were found between stilbenes and all BC risk (OR_Q4 vs. Q1_: 0.70, 95%CI: 0.56–0.89, *P*_trend_ = 0.001), the consumption of hydroxybenzaldehydes (OR_Q4 vs. Q1_: 0.75, 95%CI: 0.59–0.93, *P*_trend_ = 0.012) and hydroxycoumarins (OR_Q4 vs. Q1_: 0.73, 95%CI: 0.57–0.93; *P*_trend_ = 0.005) were also inversely associated. The intake of stilbenes, hydroxybenzaldehydes and hydroxycoumarins can contribute to BC reduction risk on all menopausal and receptor statuses.

## 1. Introduction

Vegetables and fruits contain plant secondary metabolites called polyphenols, which can be classified in more than 24 subclasses based on their chemical structure, comprising more than 5000 different individual compounds. Polyphenols can have diverse bioactive effects [1].

Polyphenol consumption could reduce the risk of cancer development through various mechanisms [2,3,4,5], protecting against DNA damage [6], blocking specific carcinogen pathways [7], inducing apoptosis [8], acting as antioxidant and anti-inflammatory agents [9], inhibiting angiogenesis [10], and/or suppressing matrix metalloproteinase secretion and tumor invasiveness [11].

Breast cancer (BC) is the leading cause of cancer death among women in the world, responsible for 25% of the total new cancer cases and 627,000 deaths in 2018 [12]. Incidence rates vary across world regions, with a higher prevalence rate in more developed areas. In Spain, the BC yearly incidence is estimated to be more than 30,000 new cases and over 6000 deaths [12].

According to the continuous update project, early menarche (before the age of 12), late menopause (after the age of 55) and not bearing children increase time exposure to estrogen and progesterone and the risk of BC [13]. This report also indicated that, although there is limited evidence on the effect of vegetables on reducing the risk of BC [13], unhealthy diets and low physical activity increase sex hormones status independently from any other BC risk factor [14,15]. In addition, some studies have reported that a few polyphenol-induced estrogen receptor (ER) responses are comparable or even superior to those induced by physiological levels of estradiol. This can be a reason why some polyphenols are still described as complete estrogen agonists and have a superior affinity for ER-β [16]. Moreover, phytoestrogens can also alter estrogen biosynthesis and decrease the concentrations of circulating levels, acting as cytochrome P450 19 (Cyp19) aromatase inhibitors, of 17β- hydroxysteroid dehydrogenases (HSD), estrone sulfatases and sulfotransferases [17]. Thus, some polyphenols, particularly phytoestrogens, may have different effects on BC risk depending on hormone receptor status.

Several epidemiological studies, focused on lignans and flavonoids, have detected a protective association with BC risk [1,18,19], but, to our knowledge, the effect of other polyphenol classes has not been assessed. Nevertheless, meta-analyses have shown that flavonoid intake effect on BC risk is not well established, considering differences between tumor characteristics such as estrogen receptor, progesterone receptor and HER2 receptor status [20,21]. 

The aim of this study was to evaluate the effect of dietary intake of all polyphenol classes (flavonoids, phenolic acids, stilbenes, lignans and others) on total BC and by and hormone receptor status in the Multi-Case-Control (MCC)-Spain study.

## 2. Materials and Methods 

**Figure 1:**
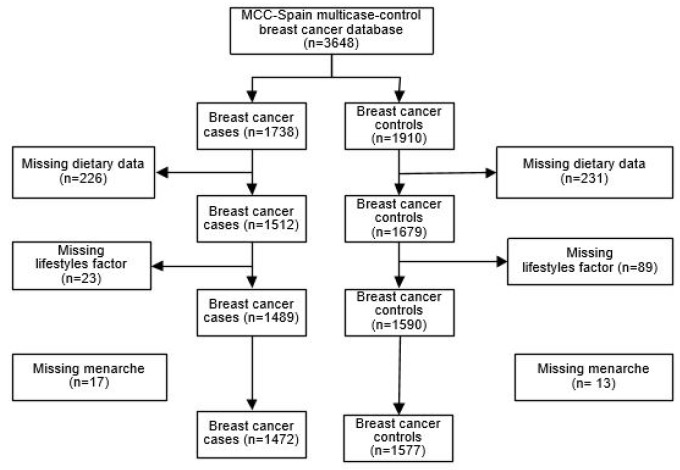
Algorithm for selection of breast cancer controls and cases in the multi-case-control (MCC)-Spain study.

MCC-Spain [22] is a population-based multi-case-control study carried out between September 2008 and December 2013 in 12 Spanish provinces. The methodology included recruiting cases and controls, as has been previously described [23]. Briefly, BC cases were women aged 20 to 85 years old and newly diagnosed with histologically confirmed BC, and were recruited from 10 of the 12 participating Spanish provinces. A single set of population-based controls were frequency-matched to cases, by age and region. Controls were randomly selected from primary care centers within catchment areas of the hospitals where the cases were recruited. As can be observed in Figure 1, the initial 3648 individuals (1738 first confirmed cases of BC and 1910 controls) were filtered by specific exclusion criteria for the statistic models—participants with no polyphenol dietary data, menopausal status, socioeconomic status, smoking status, alcohol consumption, oral contraceptive consumption (OCC), family history of BC, menarche, number of children, physical activity, and body mass index (BMI). The final database included 1577 controls and 1472 BC cases, of which 990 were hormonal receptor positive (ER+ or PR+), 249 were ERB2+ (independently of ER and PR status) and 106 were triple negative (TNBC) tumors.

### 2.1. Data Collection

Data on sociodemographic factors, lifestyle and personal/family medical history were collected with a structured computerized epidemiological questionnaire that was administered by trained personnel in a face-to-face interview [23]. Habitual dietary information of the previous year was obtained with a validated 154-item food frequency questionnaire (FFQ) [24]. 

Similarly to other studies, if a given food was a mixture of several others (e.g., “soup” or vegetable puree) the recipe was calculated (sum of ingredients). Total energy intake and nutrients were also estimated. Moreover, some questions about general dietary habits were included in the questionnaire and were used to adjust the responses to the FFQ according to Calvert et al. methodology [25].

In this study, the daily intake of four classes and 22 subclasses of polyphenols was estimated using both Phenol-Explorer food-composition database [26] and USDA food-composition databases [27]. Data on proanthocyanidins (dimers, trimers, 4–6 mers, 7–10 mers and >10-mers) were extracted from the United States Department of Agriculture (USDA) database [27], because more data were available in the USDA database than in Phenol-Explorer. 

Polyphenol intake was calculated in mg per day, using the food consumption data from the FFQ and the polyphenol content (expressed as aglycones) of each food included in the Phenol-Explorer database [28]. Data provided by the Phenol-Explorer tool were insufficient to apply retention factors in the calculation of polyphenol intake. Phenol-Explorer data provide information on polyphenol content obtained from chromatography with and without hydrolysis. Since polyphenol data using chromatography without hydrolysis has missing values, this information was completed using data on chromatography after hydrolysis.

### 2.2. Statistical Analysis

Polyphenol intake was positively correlated with total energy intake. For this reason, the contents of polyphenols were adjusted for total energy using the residuals method [29], and posteriorly categorized in quartiles according to its distribution among controls. In addition, the consumption of polyphenols was log_2_-transformed to correct right-skewedness [30] and to facilitate the interpretation of the results. 

Odds ratios (ORs) and 95% confidence interval (CI) of BC risk were calculated according to the polyphenol consumption quartiles using mixed effects logistic regression adjusted by known BC risk factors and by menopausal status and receptor status [13,31]. Adjusted ORs of Q4 vs. Q1 and 95% confidence intervals (CI) of BC according to polyphenol intake were adjusted. 

Age (y), family history of cancer (yes, no), smoking status (never-smoker, ever-smoker), consumption of nonsteroidal anti-inflammatory drugs (NSAIDs; yes, no), consumption of alcohol (0, <12, 12–47, >47 g/day), socioeconomic status (low, medium, high), BMI (<30, ≥30 kg/m^2^), physical activity (0, 0–8, 8–16, >16 METS*h/week), age of menarche (≤11, 12–14, >14 years), number of children (0, 1, 2, >2), oral contraception consumption (ever, never), hormone replacement therapy (ever, never; only for postmenopausal women) and energy intake (kcal/day) as fixed effects and province of residence as a random effect term. In a sensitivity analysis, we further adjusted the previous model for fibre (mg/d) and vitamin C (mg/d) to account for potential interactions with other bioactive nutrients also present in some polyphenol-rich foods. The age at first child and lactation were included in the first models that we carried out as in other studies, but did not generate any change in the results.

Heterogeneity of the effects by menopausal status was tested by including in the models an interaction term between polyphenol intake and menopausal status. To evaluate these associations by BC subtypes, multinomial logistic regression methods were used. These models were adjusted by the same set of variables described above, plus the province of residence. 

Given the multiple comparisons, to control the expected proportion of discoveries that are false, an FDR (false discovery rate) test through the Benjamini–Hochberg procedure was made (*p* < 0.05) (see Tables S1–S6).

Stata statistical software (Version 13, Stata Corp, College Station, TX, US) [32] was used for mixed effects logistic regression, Python Version 3.14, Python Software Foundation, Delaware, US) [33] and R (Version 3.6, R Foundation for Statistical Computing, Vienna, Austria) [34] for the extraction of polyphenol content data in each food, and the calculation of polyphenol consumption by each individual, respectively. 

## 3. Results

Characteristics of the study population and sociodemographic factors by case-control status and by menopausal status are shown in Table 1. The percentage of polyphenol input of food in all individuals studied is presented in Table 2.

**Figure 2:**
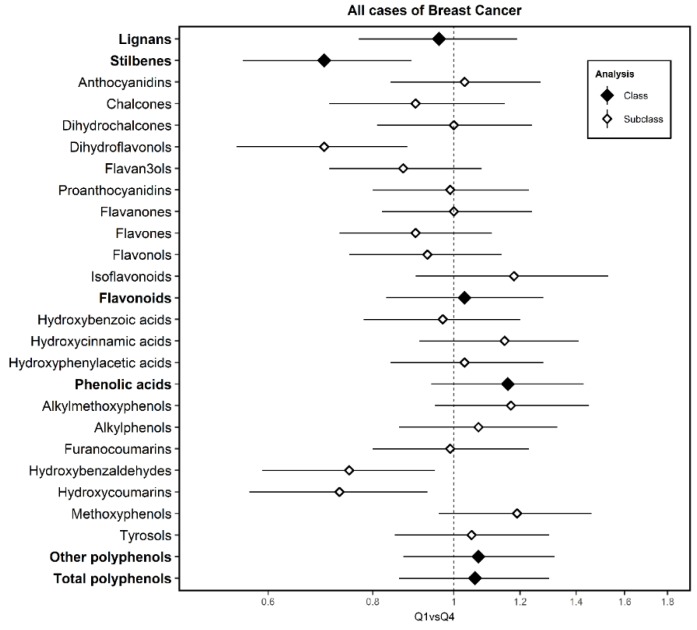
Adjusted Odds Ratios (ORs) of Quartile 4 (Q4) vs. Quartile 1 (Q1) and 95% confidence intervals (CI) of breast cancer according to polyphenol intake in the multi-case-control (MCC)-Spain study. Adjusted ORs of Q4 vs. Q1 and 95% confidence intervals (CI) of BC according to polyphenol intake. ORs were adjusted for age, socioeconomic status, BC family history, body mass index, smoking, physical activity, energy, NSAIDs, age of menarche, number of children, past alcohol intake, hormone replacement therapy and oral contraceptives consumption as fixed effects and province of residence as a random effect term.

Figure 2 shows the results of the different subclasses of polyphenols for all cases of BC (see Table S7). Total consumption of polyphenols has not been associated with BC risk (aOR_Q4 vs. Q1_ = 1.06; 95% CI = 0.86–1.30). The intake behavior of the various families is heterogeneous, ranging from the clear protective association with stilbenes (aOR_Q4 vs. Q1_ = 0.70; 95% CI = 0.56–0.89) to the probable higher risk with phenolic acids (aOR_Q4 vs. Q1_ = 1.16; 95% CI = 0.94–1.43) and without noticeable effect in the case of flavonoids, lignans and other polyphenols. As with families, a heterogeneous behavior with the various compounds is observed, highlighting the protective and statistically significant association with dihydroflavonols (aOR_Q4 vs. Q1_ = 0.70; 95% CI = 0.55–0.88), hydroxibenzaldehydes (aOR = 0.75; 95% CI = 0.59–0.95) and hydroxicoumarins (aOR_Q4 vs. Q1_ = 0.73; 95% CI = 0.57–0.93). We also observed a possible higher risk associated with high metoxyphenol intake (aOR_Q4 vs. Q1_ = 1.19; 95% CI = 0.96–1.46). In the sensitivity analysis, almost identical values were observed after additionally adjusting the multivariable model for fiber and vitamin C.

**Figure 3:**
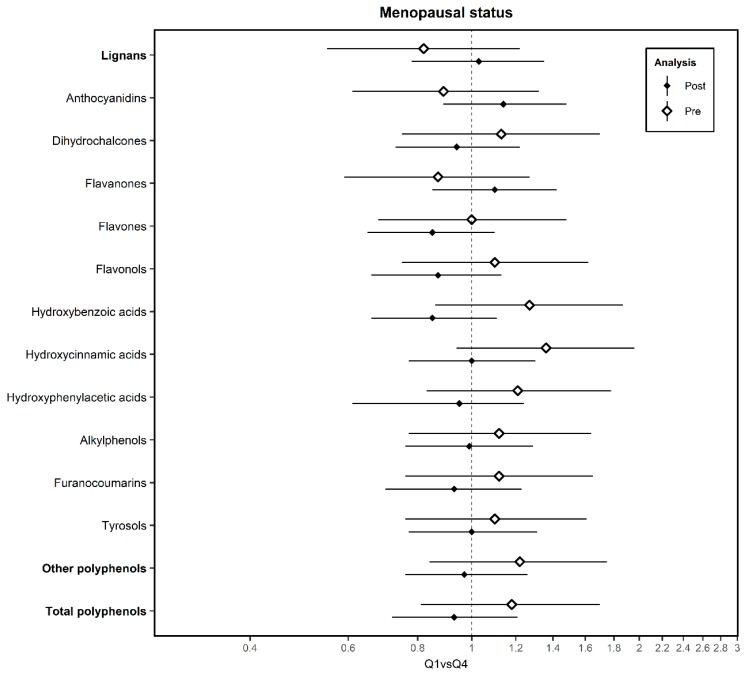
Association between estimated intake of subclasses of polyphenols with breast cancer by menopausal status, in the Multi-Case-Control (MCC)-Spain study. Post: Postmenopausal women; Pre: Premenopausal women. Adjusted ORs of Q4 vs. Q1 and 95% confidence intervals (CI) of BC by menopausal status according to polyphenol intake. ORs were adjusted for age, socioeconomic status, BC family history, body mass index, smoking, physical activity, energy, NSAIDs, age of menarche, number of children, past alcohol intake, hormone replacement therapy and oral contraceptives consumption as fixed effects and province of residence as a random effect term.

In Figure 3 we only represented the results for the polyphenols that we observed a different direction in the associations between pre- and postmenopausal women (the rest of the results are in Table S7). A notable difference has been observed for the case of isoflavonoids, in which high consumption was associated with higher risk among premenopausal women (aOR_Q4 vs. Q1_ = 1.62; 95% CI = 1.00–1.62) while no association was observed among postmenopausal (aOR_Q4 vs. Q1_ = 0.98; 95% CI = 0.71–1.35).

**Figure 4:**
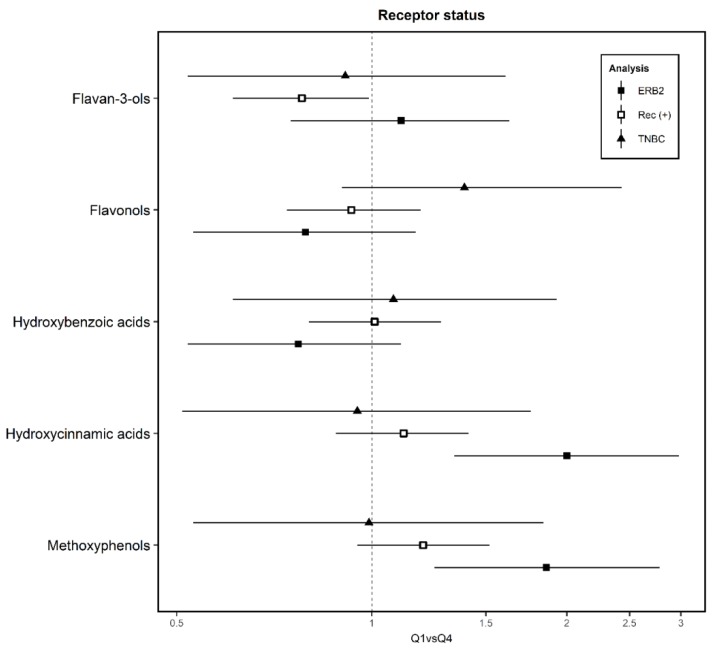
Association between estimated intake of subclasses of polyphenols with breast cancer by receptor status, in the Multi-Case-Control (MCC)-Spain study. ERB2: Erb B-2 receptor; Rec (+): hormonal receptor positive; TNBC: triple negative breast cancer. Adjusted ORs of Q4 vs. Q1 and 95% confidence intervals (CI) of BC by receptor status according to polyphenol intake. ORs were adjusted for age, socioeconomic status, BC family history, body mass index, smoking, physical activity, energy, NSAIDs, age of menarche, number of children, past alcohol intake, hormone replacement therapy and oral contraceptives consumption, and area of residence (random effects).

Regarding hormonal receptors, we highlighted only polyphenols with differences in the results (Figure 4); the rest of the results are shown in Table S8. In the case of flavan-3-ols where they behave as a protective factor against tumors with positive hormonal receptors (aOR_Q4 vs. Q1_ = 0.78; 95% CI = 0.61–0.99), flavonols as a risk factor against TNBC (aOR_Q4 vs. Q1_ = 1.39; 95% CI = 0.90–2.43) and protective against ERB (+), hydroxybenzoic acids as a possible protective association in the case of ERB (+), the higher risk of hydroxicinnamic acids for ERB (+) (aOR_Q4 vs. Q1_ = 2.00; 95% CI = 1.34–2.98) and metoxyphenols (aOR_Q4 vs. Q1_ = 1.86; 95% CI = 1.25–2.78).

## 4. Discussion

Our results suggest that the intake of total polyphenols, flavonoids, and lignans was not associated with BC risk. However, a significantly lower risk was found with stilbenes and, a probable higher risk with phenolic acid intake. Our results indicate an inverse significant association between all BC cases with dihydroflavonols, hydroxybenzaldehydes and hydroxycoumarins. Regarding the results by menopausal status, it is important to take into account the variability of the associations with dihydrochalcones, flavanones, flavonols, hydroxybenzoic acids, isoflavones with menopausal status. Moreover, polyphenol subclasses showed a protective association with hydroxycoumarins and a higher risk with isoflavonoids only for postmenopausal women. In relation to receptor status, our results showed that the intake of polyphenol subclasses such as chalcones, dihydroflavonols, hydroxybenzoic acids, stilbenes, and hydroxycoumarins could act as a protective factor in the development of BC. We found protective associations for hormonal receptor (+) BC with chalcones, dihydroflavonols, flavan-3-ols, and stilbenes. Meanwhile, chalcones, dihydroflavonols, flavonols, stilbenes, hydroxybenzaldehydes, and hydroxycoumarins were inversely associated with the risk of the Erb2 subtype, while alkylmethoxyphenols and methoxyphenols subclasses are directly associated. Finally, dihydroflavonols, stilbenes, hydroxybenzaldehydes and hydroxycoumarins were associated with a lower risk of developing TNBC.

These results are in line with those obtained by the study of Zamora-Ros et al. [28], in which the consumption of flavonoids and lignans had no significant association with BC risk. The protective association found in the case of stilbenes was also found in the study of Levi et al. [35], which indicated a reduction of BC risk with resveratrol (the main contributor of the stilbene class). It had a protective significant association taking into account resveratrol from wine and grapes together, and only from grapes, but this effect was not significant when only resveratrol from wine was taken into account, probably due to the alcohol content of wine.

In contrast, there is controversy about our results with other published studies as Feng et al. [36] and Gardeazabal [37]. The first one determined that the consumption of flavonoids was associated with lower BC risk [36]. The second one (that studied the total intake of different classes of polyphenols) did not find any significant associations between BC risk and the intake of total flavonoids, total lignans, stilbenes and total phenolic acids [37], although achieving a nonsignificant risk reduction. However, a Fink et al. [38] study found a significant inverse association for lignans but not for total flavonoid consumption. 

In relation to the different subclasses of polyphenols in all categories performed (menopausal status and receptor status) and the risk of developing BC, the heterogeneity found must be taken into account. Contrary to these results, the Fink et al. study [38] found a significant inverse association of flavonols and flavones consumption with BC risk. On the other hand, Feng et al. [36] found a protective effect of anthocyanidins, proanthocyanidins, flavanones, flavones, flavonols and isoflavones. Some other studies found a protective effect of flavonol consumption against BC risk among none-to-low alcohol drinkers compared to heavy drinkers [18,19].

Case-control studies carried out in USA [38], Mexico [39,40] and Greece [41] observed a lower risk of BC among postmenopausal women with a high intake of some flavonoid subclasses (i.e., flavones, flavanols, and flavonols). Nevertheless, results from prospective cohort studies indicated that the chemopreventive role of flavonoids and flavonols in BC carcinogenesis still remains unclear [18,19,42]. Other studies suggested that protective associations were stronger in premenopausal than in postmenopausal women [43,44], whereas Dong et al. showed the opposite [45]. Concerning isoflavones, a meta-analysis [45] concluded that in Asian countries its consumption might be associated with a lower risk of BC, probably due to the high soy intake. The same meta-analysis did not find an association between isoflavone intake and BC risk in European countries. 

This protective associations could be explained by the ability of some polyphenol subclasses to generate similar responses to estrogen (phytoestrogens), as their structure resembles the most important type of estrogen in humans and possesses hydroxyl groups and phenolic rings, necessary for binding to estrogen receptors [46]. In addition, several studies reported that some of the estrogen receptor (ER)-mediated responses induced by flavonoids are comparable, or even superior, to those induced by physiological levels of estradiol [47]. Thus, some flavonoids are still described as complete estrogen agonists and with a higher affinity for ERβ, exerting a response that opposes the proliferative effects of ERα activation [48,49]. This suggests that, at physiological levels, phytoestrogens can activate ERβ but not procancer signaling mediated by ERα (or activate it to a much lesser extent) enabling a beneficial antiproliferative effect. Regarding receptor status, two studies found no association between flavonoid intake and BC risk [28,50]. In contrast, we found a protective relation for hormonal receptor (+) BC with chalcones, dihydroflavonols, flavan-3-ols, and stilbenes. 

In our study, polyphenol classes showing a protective association against BC risk (i.e., stilbenes, dihydroflavonols, and hydroxycoumarins) seem to be mainly related to grape-wine consumption. Even though high alcohol consumption is related to an increase of BC risk, in our database, 85.6% of controls and 84.6% of cases were distributed in 0 and 0–12 g/day of alcohol consumption. The protective association for low dose wine consumption with BC risk could be explained by several mechanisms, as wine contains high concentrations of many polyphenols, among them resveratrol, the most well-known stilbene. An in vitro study has proved the antiproliferative activity of these compounds on different BC cell lines, indicating that at nM or even at pM plasma concentrations, obtained after moderate stilbene ingestion, stilbenes have a protective effect against BC risk [51]. 

However, our results must be interpreted with caution, given that they are not exempt from limitations. Since the present study is based on a case-control study design, therefore results are prone to selection and recall bias. The MCC recruited the controls from the general population (population-based case-control study), so selection bias was reduced. BC patients did not usually change their diet before diagnosis; although, recall bias is difficult to control in retrospective studies. Moreover, it is possible that cases applied as a lower consumption of alcohol than the reality, and as a consequence these flavonoids appear as protective features [52]. The difficulty in estimating of polyphenol content in foods between databases (USDA, Phenol-Explorer), the losses of polyphenols during cooking or processing, and the accuracy of FFQ, limit the precision of associations found in epidemiological studies. Polyphenols are extensively metabolized within the human body after ingestion, both at the hepatic and intestinal level, which vary widely among individuals and could affect the bioavailability of polyphenols. It must be taken into account that a part of the variability between the results from different studies could be attributable to the heterogeneity of the local dietary patterns as well as to the variations of polyphenol content in foods that can vary according to plant species, environmental conditions, or geographic and storage conditions [53]. Finally, dietary polyphenols are consumed simultaneously with other nutrients and compounds. Although we have adjusted for some of the most relevant ones found in polyphenolic-rich foods (such as alcohol, fiber and vitamin C), the possible confounding/interactions with other nutrients/compounds cannot be ruled out.

This is the first study to carry out an analysis including all polyphenol classes and subclasses by menopausal and receptor status. Several reviews [2,3,4,5] summarized the existing evidence about the association between cancer risk and polyphenol intake [53,54,55], suggesting many potential beneficial effects. Flavonoids are the most studied polyphenol class, while other polyphenol classes and compounds, which are also widely consumed by the European population [56], have been rarely investigated. This lack of epidemiologic studies exploring the relationships between the intake of polyphenol subclasses and BC risk by menopausal and receptor status precluded us from comparing our results with others. In addition, models were calculated in quartiles and log2 to facilitate the comparison with previous studies. An FDR test was used to control false discoveries. Phenol-Explorer was built including all the available information about polyphenol contents in Phenol-Explorer, with a mix of extracted data from chromatography, chromatography after hydrolysis, and USDA data. Overall, our data on total polyphenol intake in the Spanish population sampled by the MCC-Spain study are consistent with previous reports [56], which speak in favor of the accuracy of our estimations of polyphenol intake. Finally, to our knowledge, this is the first study that explores the association of a high intake of polyphenols with BC risk including a wide variety of classes and subclasses and taking into account menopausal and hormonal receptor status. 

## 5. Conclusions

The present study suggests that there might be a high variability in the results obtained when exploring the effect of polyphenols on BC risk if classes and subclasses of polyphenols or menopausal and hormonal receptor status are taken into account. Therefore, it is important that future studies on this topic include such information. 

## Figures and Tables

**Table 1:** Distribution of lifestyle by cases, controls of breast cancer and menopausal status.

Variables		Controls (*N* = 1577)	Breast Cancer Cases (*N* = 1472)	Premenopausal (*N* = 1006)		Postmenopausal (*N* = 2043)	
				Control (*N* = 471)	Cases (*N* = 535)	Control (*N* = 1106)	Cases (*N* = 937)
Socioeconomic status	High (%)	281 (17.78)	238 (16.19)	143 (30.36)	129 (24.11)	138 (12.48)	109 (11.63)
	Medium (%)	8170 (51.90)	786 (53.39)	265 (56.26)	335 (62.62)	552 (49.51)	451 (48.13)
	Low (%)	479 (30.32)	448 (30.42)	63 (13.38)	71 (13.27)	416 (37.61)	377 (40.23)
Smoking status (%)	Yes	639 (40.51)	660 (44.92)	261 (55.41)	328 (61.31)	378 (34.18)	332 (35.43)
	No	938 (59.49)	812 (55.08)	210 (44.59)	207 (38.69)	728 (65.82)	605 (64.57)
Family history of breast cancer (%)	Yes	145 (9.24)	212 (14.36)	25 (5.31)	77 (14.39)	120 (10.85)	135 (14.41)
	No	1432 (90.76)	1260 (85.64)	446 (94.69)	458 (85.61)	986 (89.15)	802 (85.59)
NSAID (%)	Yes	807 (51.14)	656 (44.58)	240 (50.96)	235 (43.93)	567 (51.27)	421 (44.93)
	No	770 (48.86)	816 (55.42)	231 (49.04)	300 (56.07)	539 (48.73)	516 (55.07)
BMI (kg/m^2^)	<30 kg/m^2^	1313 (83.29)	1214 (82.38)	417 (88.54)	491 (91.78)	896 (81.01)	723 (77.16)
	≥30 kg/m^2^	264 (16.71)	268 (17.62)	54 (11.46)	44 (8.22)	210 (18.99)	214 (22.84)
Alcohol consumption (g/day)	0 g/day	401 (25.57)	357 (24.46)	97 (20.59)	97 (18.13)	304 (27.49)	260 (27.75)
	0–12 g/day	965 (61.08)	887 (60.09)	320 (67.94)	367 (69.60)	645 (58.32)	520 (55.50)
	12–47 g/day	192 (12.15)	202 (13.69)	50 (10.62)	62 (11.59)	142 (12.82)	140 (14.94)
	>47 g/day	19 (1.2)	26 (1.76)	4 (0.85)	9 (1.68)	15 (1.36)	17 (1.81)
Physical activity	0 METS*h/week	592 (37.59)	629 (42.62)	207 (43.95)	242 (45.23)	385 (34.81)	387 (41.30)
	0–8 METS*h/week	257 (16.33)	229 (15.51)	86 (18.26)	101 (18.88)	171 (14.46)	128 (13.66)
	8–16 METS*h/week	227 (14.37)	190 (12.94)	66 (14.01)	71 (13.27)	161 (14.56)	119 (12.70)
	>16 METS*h/week	501 (31.71)	424 (28.93)	112 (23.78)	121 (22.62)	389 (35.17)	303 (32.34)
Oral contraceptive consumption	never	792 (50.19)	763 (51.83)	139 (29.51)	178 (33.27)	653 (59.04)	585 (62.43)
	ever	785 (49.81)	709 (48.17)	332 (70.49)	357 (66.73)	453 (40.96)	352 (37.57)
Hormone replace therapy	never	1403 (88.99)	1335 (90.65)	-	-	933 (84.36)	801 (85.49)
	ever	121 (7.66)	104 (7.05)	470 (99.79)	534 (99.81)	120 (10.85)	103 (10.99)
	not known	53 (3.35)	33 (2.3)	1 (0.21)	2 (0.20)	53 (4.79)	33 (3.52)
Number of children	0	303 (19.18)	309 (20.93)	132 (28.03)	137 (25.61)	171 (15.46)	171 (18.25)
	1	251 (15.95)	278 (18.83)	114 (24.20)	136 (25.42)	137 (12.39)	142 (15.15)
	2	629 (39.94)	592 (40.31)	183 (38.85)	215 (40.19)	446 (40.33)	377 (40.23)
	>2	394 (24.94)	294 (19.92)	42 (8.92)	47 (8.79)	352 (31.83)	247 (26.36)
Menarche	<11 years old	81 (5.25)	94 (6.37)	23 (4.88)	31 (5.79)	58 (5.24)	63 (6.72)
	12–14 years old	1305 (82.66)	1211 (82.25)	413 (87.69)	460 (85.98)	892 (80.65)	751 (80.15)
	>14 years old	191 (12.09)	167 (11.38)	35 (7.43)	44 (8.22)	156 (14.10)	123 (13.13)

**Table 2:** Main foods that contribute more to each subclass of polyphenol.

Polyphenol Class	Subclass	Compound	Food Sources *	Mean Intake (g/d)
Lignans		1-Acetoxypinoresinol, Pinoresinol, 7-Hydroxymatairesinol, 7-Oxomatairesinol, Conidendrin, Cyclolariciresinol, Isolariciresinol, Lariciresinol, Lariciresinol-sesquilignan, Matairesinol, Medioresinol, Pinoresinol, Secoisolariciresinol, Secoisolariciresinol-sesquilignan, Syringaresinol	Olive oil (94.8%), Gazpacho (5.2%)	2.92
Stilbene		d-Viniferin, Pallidol, Piceatannol, Resveratrol	Red wine (76.1%), Strawberry (7.7%), Rosé/White wine (7.5%), Grapes (5.8%), Lentils (1.1%), Chocolate (1.1%)	0.85
Flavonoids				143.38
Anthocyanins		Cyanidin, Delphinidin, Malvidin, Pelargonidin, Peonidin, Petunidin, Pinotin A, Vitisin A	Sweet cherry (39.6%), Strawberry (21.0%), Plum (11.3%), Grapes (10.6%), Olives (9.6%), Red wine (6.5%)	19.42
Chalcones		Xanthumol	Beer Ale (95%), Beer alcohol free (5%)	0.002
Dihydrochalcones		Phloretin, 3-Hydroxyphloretin	Apple (73.4%), Nonorange juice (26.6%)	1.05
Dyhydroflavonols		Dihydroquercetin	Red wine (95%), Rosé/White wine (5%)	0.83
Flavanols		(-)-Epicatechin, (-)-Epigallocatechin, (+)-Catechin, (+)-Epicatechin-(2a-7)(4a-8)-epicatechin, (+)-Gallocatechin, Cinnamtannin A2	Cocoa powder (58.1%), Chocolate (13.1%), Broad bean seed (5.6%), Plum (5.3%), Red Wine (5.3%), Apple (3.5%), Sweet cherry (1.7%), Persimmon/Custard apple (1.5%), Strawberry (1.0%), Grapes (1.0%)	23.10
Flavanones		6-Prenylnaringenin, 8-Prenylnaringenin, Eriodictyol, Hesperetin, Isosakuranetin, Isoxanthohumol, Naringenin	Orange pure juice (72.2%), Non-orange pure juice (24.1%), Red wine (1.5%)	43.33
flavones		Apigenin, Chrysoeriol, Diosmetin, Luteolin, Nobiletin, Sinensetin, Tangeretin, Tetramethylscutellarein	Globe artichoke (62.9%), Celery (18.1%), Olives (11.7%), Orange pure juice (2.0%), Vegetable soup (1.3%), Sweet pepper green (1.1%), Lettuce (1.1%)	4.00
Flavonols		3,7-Dimethylquercetin, 3-Methoxynobiletin, 5,3’,4’-Trihydroxy-3-methoxy-6:7-methylenedioxyflavone, 5,4’-Dihydroxy-3,3’-dimethoxy-6:7-methylenedioxyflavone, 6,8-Dihydroxykaempferol, Ferulic acid, Isorhamnetin, Jaceidin, Kaempferol, Morin, Myricetin, Patuletin, Quercetin, Spinacetin	Swiss chard (23.2%), Common beans (18.9%), Endive (8.0%), Olives (7.9%), Chocolate (7.8%), Asparagus (7.2%), Chickpea/Common beans (5.8%), Lettuce (3.7%), Red wine (3.2%), Plum (2.2%), Green bean (2.0%), Onion (1.8%), Apple (1.4%), Grapes (1.1%)	23.10
Isoflavonoids		Biochanin A, Daidzein, Genistein, Glycitein, Formononetin	Soy milk (93.9%), Common Beans (4.3%), Chickpea/Common beans (1.3%)	2.26
Phenolic acids				163.85
Hydroxybenzoic acids		Valoneic acid dilactone, 2,3-Dihydroxybenzoic acid, 2,4-Dihydroxybenzoic acid, 2,6-Dihydroxybenzoic acid, 2-Hydroxybenzoic acid, 3,5-Dihydroxybenzoic acid, 3-Hydroxybenzoic acid, 4-Hydroxybenzoic acid, Benzoic acid, Ellagic acid, Gallagic acid, Gallic acid, Gentisic acid, Protocatechuic acid, Syringic acid, Vanillic acid	Olives (44.1%), Red wine (19.4%), Non-orange pure juice (11.8%), Strawberry (6.4%), Nuts (5.7%), Rosé/White wine (2.1%), Beer Ale (1.9%), Banana (1.6%), Lentils (1.7%)	14.47
Hydroxycinnamic acids		Caffeic acid, Caffeoyl aspartic acid, Cinnamic acid, Ferulic acid, Hydroxycaffeic acid, m-Coumaric acid, o-Coumaric acid, p-Coumaric acid, Sinapic acid	Coffee (36.3%), Globe artichoke (16.4%), Olives (11.1%), Plum (7.2%), Sweet cherry (7.0%), cocoa powder (5.9%), Red wine (2.1%), Apple (2.0%), chocolate (1.9%), Peach/Apricot (1.5%), Carrot (1.5%), Potato (1.2%), Grapes (1.0%)	149.37
Hydroxyphenylacetic acids		3,4-Dihydroxyphenylacetic acid, 4-Hydroxyphenylacetic acid, Homovanillic acid, Homoveratric acid, Methoxyphenylacetic acid	Olives (96.3%), Red wine (2.3%)	0.55
Other polyphenols				13.12
Alkylmethoxyphenols		4-Vinylguaiacol	Coffee (96.4%), Beer Ale (3.6%)	0.72
Alkylphenols		3-Methylcatechol, 4-Ethylcatechol, 4-Methylcatechol, 3-Methylcatechol, 4-Vinylphenol	Coffee (83.4%), Cocoa powder (14.8%), Beer (1.8%)	0.1
furanocoumarins		Bergapten, Isopimpinellin, Psoralen, Xanthotoxin	Celery (91.6%), Non-orange pure juice (8.4%)	0.03
Hydroxybenzaldehydes		Protocatechuic aldehyde, Syringaldehyde, Vanillin	Red wine (67.2%), Cocoa powder (9.5%), Cognac/Rum/Whisky (7.8%), Olives (4.7%), Rosé/White wine (4.7%), Sherry (3.3%), Cider/Champagne (1.1%)	0.17
Hydroxycoumarins		4-Hydroxycoumarin, Esculetin, Mellein, Scopoletin, Umbelliferone	Rosé/White wine (58.8%), Beer Ale (22.8%), Cocoa powder (11.4%), Sherry (7.0%)	0.04
Methoxyphenols		Guaiacol	Coffee (100%)	0.10
Tyrosol		Hydroxytyrosol acetate (4-DHPEA-AC), Hydroxytyrosol, Oleoside 11-methylester, Tyrosol acetate (p-HPEA-AC), Tyrosol	Olives (83.2%), Olive oil (11.9%), Red wine (2.4%), Cider/Champagne (1.0%)	11.98

* Food sources that contribute >1%.

## Supplementary Materials

The following are available online at https://www.mdpi.com/2072-6643/12/4/994/s1, Table S1: Results of Benjamini-Hochberg in all breast cancer cases according to polyphenol subclass intake in the MCC-Spain, Table S2: Results of Benjamini-Hochberg in all premenopausal cases according to polyphenol subclass intake in the MCC-Spain, Table S3: Results of Benjamini-Hochberg in all postmenopausal cases according to polyphenol subclass intake in the MCC-Spain, Table S4: Results of Benjamini-Hochberg in hormonal receptor (+) according to polyphenol subclass intake in the MCC-Spain, Table S5: Results of Benjamini-Hochberg in erb-2 according to polyphenol subclass intake in the MCC-Spain, Table S6: Results of Benjamini-Hochberg in triple-negative breast cancer according to polyphenol subclass intake in the MCC-Spain, Table S7: Association between estimated intake of subclasses of flavonoids, phenolic acids, and other polyphenols with breast cancer among all BC cases, pre- and postmenopausal women, in the MCC-Spain case-control database; Table S8: Association between estimated intake of subclasses of flavonoids, phenolic acids, and other polyphenols by hormonal receptor, in the MCC-Spain case-control database.
